# UDP-N-Acetylglucosamine 2-Epimerase/N-Acetylmannosamine Kinase (GNE) Binds to Alpha-Actinin 1: Novel Pathways in Skeletal Muscle?

**DOI:** 10.1371/journal.pone.0002477

**Published:** 2008-06-18

**Authors:** Shira Amsili, Hagit Zer, Stephan Hinderlich, Sabine Krause, Michal Becker-Cohen, Daniel G. MacArthur, Kathryn N. North, Stella Mitrani-Rosenbaum

**Affiliations:** 1 Goldyne Savad Institute for Gene Therapy, Hadassah Hebrew University Medical Center, Jerusalem, Israel; 2 Biacore Laboratory, Interdepartmental Equipment Unit, Institute of Life Sciences, The Hebrew University of Jerusalem, Jerusalem, Israel; 3 Charité–Universitätsmedizin Berlin, Campus Benjamin Franklin, Berlin, Germany; 4 Institut für Biochemie und Molekularbiologie, Berlin-Dahlem, Berlin, Germany; 5 Technische Fachhochschule Berlin, Fachbereich Life Sciences & Technology, Berlin, Germany; 6 Friedrich-Baur-Institut, Neurologische Klinik und Poliklinik, Ludwig-Maximilians-Universität, München, Germany; 7 Discipline of Pediatrics and Child Health, Faculty of Medicine, Institute for Neuromuscular Research, The Children's Hospital at Westmead, University of Sydney, Sydney, Australia; Hospital Vall d'Hebron, Spain

## Abstract

**Background:**

Hereditary inclusion body myopathy (HIBM) is a rare neuromuscular disorder caused by mutations in *GNE*, the key enzyme in the biosynthetic pathway of sialic acid. While the mechanism leading from *GNE* mutations to the HIBM phenotype is not yet understood, we searched for proteins potentially interacting with GNE, which could give some insights about novel putative biological functions of GNE in muscle.

**Methodology/Principal Findings:**

We used a Surface Plasmon Resonance (SPR)-Biosensor based assay to search for potential GNE interactors in anion exchanged fractions of human skeletal muscle primary culture cell lysate. Analysis of the positive fractions by *in vitro* binding assay revealed α-actinin 1 as a potential interactor of GNE. The direct interaction of the two proteins was assessed *in vitro* by SPR-Biosensor based kinetics analysis and in a cellular environment by a co-immunoprecipitation assay in GNE overexpressing 293T cells. Furthermore, immunohistochemistry on stretched mouse muscle suggest that both GNE and α-actinin 1 localize to an overlapping but not identical region of the myofibrillar apparatus centered on the Z line.

**Conclusions/Significance:**

The interaction of GNE with α-actinin 1 might point to its involvement in α-actinin mediated processes. In addition these studies illustrate for the first time the expression of the non-muscle form of α-actinin, α-actinin 1, in mature skeletal muscle tissue, opening novel avenues for its specific function in the sarcomere. Although no significant difference could be detected in the binding kinetics of α-actinin 1 with either wild type or mutant GNE in our SPR biosensor based analysis, further investigation is needed to determine whether and how the interaction of GNE with α-actinin 1 in skeletal muscle is relevant to the putative muscle-specific function of α-actinin 1, and to the muscle-restricted pathology of HIBM.

## Introduction

Hereditary inclusion body myopathy (HIBM) is a unique neuromuscular disorder characterized by adult-onset, slowly progressive distal and proximal muscle weakness, presenting with an unusual feature, the sparing of the quadriceps. HIBM fibers have typical muscle pathology, including cytoplasmic rimmed vacuoles and cytoplasmic or nuclear filamentous inclusions composed of tubular filaments [1]. The disease is particularly common in the Jewish Persian community (with a prevalence of 1 in 1 500), and has been described also worldwide in non-Jewish families, particularly in Japan [2]. The *GNE* gene, encoding the bi-functional enzyme UDP-*N*-acetylglucosamine 2-epimerase/*N*-acetylmannosamine kinase (UDP-GlcNAc 2-epimerase/ManNAc kinase), is mutated in all HIBM patients studied to date. A single homozygous missense mutation, M712T, located at exon 12 of the gene, within its kinase domain, was identified in all Persian and other Middle Eastern Jewish and non-Jewish HIBM patients [3]. Other missense mutations in *GNE* have been identified in HIBM patients worldwide [4]–[7]. GNE catalyzes two sequential steps in the biosynthetic pathway of sialic acid [8], the most abundant terminal monosaccharide on glycoconjugates of eukaryotic cells [9]. The process by which mutations in this enzyme lead to the disease is not yet understood, and the issue of hyposialylation in HIBM muscles is still not resolved [10]–[13]. To find out whether GNE has other yet unknown biological functions in muscle tissue, which could be involved in the pathogenesis of HIBM, we tried to identify potential partners interacting with GNE. Such interactions could give some clue about novel pathways involving GNE, besides its known function in sialylation, and as such, could be evaluated for potential involvement in HIBM pathophysiology. We used an optical SPR-biosensor (Surface Plasmon Resonance) system, BIAcore [14], [15], to test GNE's interactions. This analysis, followed by in vitro binding assay and mass spectrometry, led to the identification of two potential GNE binding proteins. Using kinetics BIAcore analysis, co-IP and confocal microscopy, we show that one of them, α-actinin 1, interacts with GNE both *in vitro* and *in vivo.*

## Materials and Methods

Unless otherwise stated, all chemicals were obtained from SIGMA (St. Louis, MO, USA). Cell culture media were obtained from Biological Industries (Beit Haemek, Israel).

### GNE proteins expression and purification

*GNE* cDNAs were generated from total RNA isolated from lymphoblastoid cell lines derived from a healthy individual and from an HIBM patient carrying the M712T mutation in *GNE*, as described [10]. The cDNA sequences were cloned into pFASTBACAHTa expression vectors, expressed in insect cells and purified as described [10]. Freshly purified His-tagged wild type (WT) and mutant GNE proteins were bound to the BIAcore chip or used for *in vitro* binding assay.

### Cell lysis

A previously established and well characterized skeletal muscle primary cell culture [16], derived from deltoid biopsy of a 46 years old healthy male donor, was used in this study. Cells at passage 8 were grown till sub-confluency in 75 cm^2^ flasks, treated with trypsin, collected and washed twice in ice-cold PBS. Cell pellets were suspended in hypotonic ice-cold lysis buffer (100 µl/10^6^ cells; 10 mM NaPi buffer pH 8, 0.1 mM EDTA, 0.1 mM DTT, 1 mM PMSF, 17 µg/ml Aprotinin, 10 µg/ml leupeptin, 1 mM Vanadate, 20 Mm β-Glycerophosphate), incubated on ice for 30′, lysed by 20 strokes through a 26 gauge needle, and centrifuged (14,000 rpm for 30′ at 4°C). Protein concentration was determined using Bradford Reagent (SIGMA). Fresh protein lysate was used on the same day for anion exchanged chromatography.

### Anion exchanged chromatography

Total protein lysate (15 mg) was diluted with buffer A (20 mM KPO4 pH 8.0) to 4.5 ml. Anion exchange chromatography was performed on an AKTA Explorer with a 1 ml Resource 30Q column (GE Healthcare–Amersham Pharmacia, Uppsala, Sweden). Sample was loaded on a column, washed with 25 ml buffer A, eluted in 20 ml gradient 0–100% of buffer B (20 mM KPO4 pH 8.0 + 1 M NaCl) and collected in fractions of 1.2 ml. After elution, fractions with more than 0.2 M NaCl were dialyzed against 0.1 M NaCl buffer. Freshly prepared fractions were used for BIAcore analyses.

### BIAcore analysis

All experiments were carried out using BIAcore 3000 (BIAcore, Uppsala, Sweden) and sensor chip CM5 (BIAcore), at 25°C. For activating the chip, EDC/NHS amine coupling protocol was used according to BIAcore protocol (www.biacore.com). WT and mutant GNE proteins (100 µg/ml), in 10 mM acetate pH 6, were immobilized to give about 3000 RU in the analyte identification experiments and up to 600 RU in the kinetics experiments. The running buffer used was Na-phosphate buffer (10 mM NaPi pH 7.5, 100 mM NaCl, 0.1 mM EDTA, 0.1 mM DTT) for the identification experiments, and Tris buffer (20 mM Tris pH 7.5, 100–300 mM NaCl, 0.1 mM DTT, 2 mM CaCl_2_) for the kinetics. The flow rates were 10 µl/min and 30 µl/min respectively. Regeneration of the chip was done with 10 µl 3 M NaCl in 100 mM NaOH. BIAevaluation software version 4.1 was used to evaluate the results, and 1∶1 Langmuier model to fit the experimental results in order to calculate the affinity and kinetics constants.

### In vitro binding assay

Anion exchange fractions showing interaction activity in the BIAcore analysis were pooled together (300 µl total volume) and incubated in a 360° rotation shaker for 2 h at 4°C with 30 µl of Ni–NTA beads (Qiagen, Hilden, Germany) pre-bound to freshly-extracted His-tagged GNE, as described (10). The beads were washed three times with NaPi buffer (10 mM NaPi pH 7.5, 0.1 mM EDTA, 0.1 mM DTT, 100 mM NaCl), resuspended in sample buffer (62.5 mM Tris pH 6.8, 10% glycerol, 5% β-ME, 3% SDS, BPB) and eluted by 5′ incubation at 100°C. Eluted samples were separated by 7–15% gradient SDS-PAGE and the gel stained with GelCode blue stain reagent (Pierce, Rockford, IL, USA), according to the manufacturer instructions. Protein bands were cut from the gel for MS analysis.

### Mass spectrometry analysis

Reduction, alkylation and trypsinization steps were carried out in the gel as usual [17]. After extraction of the peptides from the gel with 60% CH3CN 1% CHOOH solution, and evaporation to dryness, the samples were rehydrated with 1 µl of CH3CN 1% CHOOH solution, and then diluted with 9 µl of 1% CHOOH. The peptide mixtures were either solid phase extracted with C18 resin filled tip (ZipTip; Milipore, Billerica, MA, USA) and nanosprayed into the Qtof2 MS system in 50% CH3CN 1% CHOOH solution, or injected to 0.75 µl C18 column on a capillary HPLC system (CapLC; Waters, Milford, MA, USA) coupled to the MS. MS/MS was carried out with Qtof2 (Micromass, Manchester, UK) using nanospray attachment [18]. Data analysis was done using the biolynx package V4.0 (Micromass), and database searches were performed with the Mascot package (Matrix Science, London, UK). Precursor-ion mass tolerance 1.2, fragment-ion mass tolerance 0.6, one missed cleavage, modifications allowed: Carbamidomethyl (C), Oxidation (M), Propionamide (C). Ions score is −10*Log(P), where P is the probability that the observed match is a random event. Individual ions scores >55 indicate identity or extensive homology (p<0.05). Protein scores are derived from ions scores as a non-probabilistic basis for ranking protein hits. The database searched was NCBInr 20060831 (3894824 sequences; 1340658132 residues). Five unique peptides were identified matching several members of the α-actinin family (actinin1, 2, 3, 4); nevertheless only one member of the family, α-actinin 1, aligned to each of the five peptides with 100% identity.

### LDH-B and α-actinin 1 recombinant proteins

Full length placental α-actinin 1 [19], cloned into the 6xHis pET-15b vector (Novagen, Darmstadt, Germany), was a gift of Dr. Bankston, the Burnham Institute, La Jolla, CA, USA. Transformation into BL21(DE3)pS^+^ E. coli cells and purification of the protein were performed as described [20]. Recombinant chicken LDH-B protein was purchased from ProSpec-Tany TechnoGene (Rehovot, Israel).

### Overexpression of FLAG-GNE in 293T cells

The cDNA sequence of human *GNE* (NM_005476.3) was cloned into N-terminal 3XFLAG-CMV-10 expression vector (SIGMA) according to standard procedures and transfected into human embryonic kidney 293T cells by Lipofectamine2000 (Invitrogen, Carlsbad, CA, USA) according to the manufacturer instructions. Transfected cells were selected in growth medium (DME medium containing 10% FCS, 584 µg/µl Glutamin, 20 U/ml penicillin, 20 µg/µl streptomycin) containing 400 µg/ml Geneticin (G-418; Gibco, Paisley, UK) for 10 days. The selected stably-transfected 293T cells (293T-FG) were grown in selection medium. Expression of the FLAG-tagged GNE protein in the transfected cells was verified by Western blot analysis, as described below.

### Co-Immunoprecipitation (co-IP)

FLAG-GNE transfected 293T cells (293T-FG) and untransfected (UT) control cells were grown in 75 cm^2^ flasks till sub-confluency (∼70%), crosslinked or not crosslinked with DSP (Pierce) according to the manufacturer instructions, collected and lysed as described above for muscle cells (without DTT and EDTA). Lysates (900 µg of total protein) were first incubated with 50 µl mouse ExactaCruz C IP matrix (Santa Cruz Biotechnology, Santa Cruz, CA, USA) for pre-clearing, for 30′ at 4°C, and then incubated overnight at 4°C in a 360° rotation shaker with anti-FLAG M2 mouse monoclonal antibody (10 µg/ml, SIGMA) pre-bound to 50 µl of mouse ExactaCruz C IP matrix. After washing the beads twice by 5′ rotation in PBS, immunoprecipitated proteins were eluted in sample buffer containing β-ME, as described above. As a control for successful crosslinking, 5 µg of lysate samples were dissolved in SDS-sample without β-ME.

### Western blot analysis

For Western blot analysis, lysate aliquots and eluates of co-IP were separated by 7.5% SDS–PAGE and blotted according to standard procedures. After overnight blocking in 5% BSA/PBS-Tween (0.1%), membrane was incubated overnight at 4°C with rabbit polyclonal anti α-actinin (H-300) primary antibody (1∶500; Santa Cruz Biotechnology) in 5% BSA/PBS-Tween (0.1%). Detection was performed with rabbit HRP-conjugated ExactaCruz C detection reagent (1∶1,000; Santa Cruz Biotechnology). FLAG-GNE was detected on the same membrane using anti-FLAG M2 mouse monoclonal primary antibody (SIGMA), and secondary HRP-conjugated goat anti mouse antibody (Jackson ImmunoResearch, West Grove, PA, USA). Protein expression was visualized using the SuperSignal West Pico Chemiluminescent Substrate (Pierce).

### Immunofluorescence staining of C2C12 cells

Mouse C2C12 myoblasts were cultured in 40% high-glucose Dulbecco's modified eagle medium and 40% F-12 Nutrient Mixture (HAM) with L-glutamine, supplemented with 10% fetal bovine serum and 10% horse serum. For differentiation, the cells were cultured in 48.5% high-glucose Dulbecco's modified eagle medium and 48.5% F-12 Nutrient Mixture (HAM) with L-glutamine, supplemented with 3% horse serum. C2C12 cells grown on Thermanox coverslips (Nunc) were fixed and permeabilized in PBS containing 3% paraformaldehyde and 0.1% triton X-100 for 20 minutes at room temperature. Samples were washed in PBS three times, then blocked with 2% BSA in PBS for 10 min and incubated for 1 h at room temperature with a polyclonal antibody (pAb) α-actinin-1 (3A2; 1∶100; Courtesy Prof Alan Beggs) [21]. After washing in PBS, cells were incubated 30′ at RT with the secondary antibody, Cy3-conjugated goat anti-rabbit IgG (1∶250; Jackson ImmunoResearch Laboratories, West Grove, PA), diluted in 2% BSA/PBS.

### Fixation and Immunostaining of Stretched Mouse Muscle

Freshly-harvested mouse spinalis muscle was lightly stretched in the longitudinal direction by clamping the opposite ends of the muscle onto a piece of cardboard (covered with aluminium foil). The muscle was fixed in 3% paraformaldehyde for 10 minutes at room temperature, covered in Tissue-tek and snap-frozen in liquid nitrogen-cooled isopentane. The muscle was stored in liquid nitrogen until required. Frozen muscle sections (5 µm) were blocked in 2% bovine serum albumin (BSA) in phosphate-buffered saline (PBS) for 10 minutes. After rinsing in PBS, sections were incubated overnight at 4°C in primary antibody. Primary antibodies were monoclonal antibody α-actinin-2 (1∶400; Sigma, St. Louis, MO), polyclonal antibody (pAb) α-actinin-1 (3A2; 1∶100; Courtesy Prof Alan Beggs) and polyclonal antibody GNE (1∶100, Courtesy Dr Sabine Krause) [22]. After washing in PBS, sections were blocked as mentioned above and then incubated for 3 hours at room temperature in secondary antibodies. Secondary antibodies were Cy3-conjugated goat anti-mouse IgG (1∶250; Jackson ImmunoResearch Laboratories, West Grove, PA) and Alexa Fluor 488 goat anti-rabbit IgG (1∶200; Molecular Probes, Eugene, OR). Sections were washed by immersion in PBS for 30 min and mounted on 22×50 mm^2^ glass coverslips using Fluorsave mounting reagent (Calbiochem, SanDiego, CA).

### Microscopic analysis

Confocal photographs were taken by Leica TCS SP2 Scanning Confocal Microscope equipped with HCX Plan Apo (PH3) 40×/1.25 and 63×/1.32 oil immersion objective lenses.. GelMount (SIGMA) imaging medium was used. All pictures were taken at RT.

## Results

### Biacore analysis of muscle cell extract on GNE proteins

**Figure 1:**
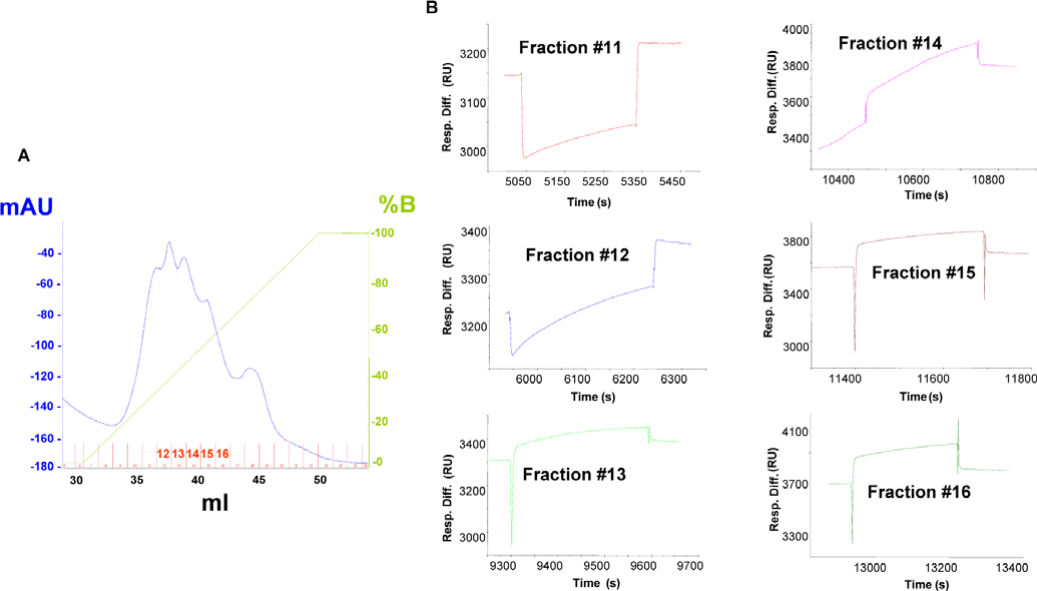
WT-GNE interacts with proteins within anion-exchanged fractions of muscle cell lysate. [A] Anion exchange chromatography (0–1 M NaCl) of cell lysate (15 mg total protein content) extracted from human skeletal muscle primary culture cells. The numbers of the positive fractions appear in red on the X axis. [B] BIAcore sensorgrams of the interaction of WT-GNE with anion exchange fractions #11–16 [Resp. Diff., response difference; RU, response units; B, buffer B (see Materials and methods)].

A freshly prepared lysate from human primary skeletal muscle culture cells (15 mg total protein content) was fractionated by anion-exchange chromatography to produce 26 fractions (Figure 1A). Interaction between GNE and the different fractions was tested using SPR technology. Freshly prepared normal and mutant (M712T) GNE proteins were immobilized on a CM5 chip using an amine coupling protocol. 50 µl of each of the fractions were injected over the immobilized chip. Binding signals were obtained, in fractions #12–16 which were eluted in 340–620 mM NaCl (Figure 1B). Similar results were obtained with the mutant GNE protein. Two pools of positive fractions (#11–13 and #14–16) were further analyzed by *in vitro* binding assay.

### In vitro binding assay

**Figure 2:**
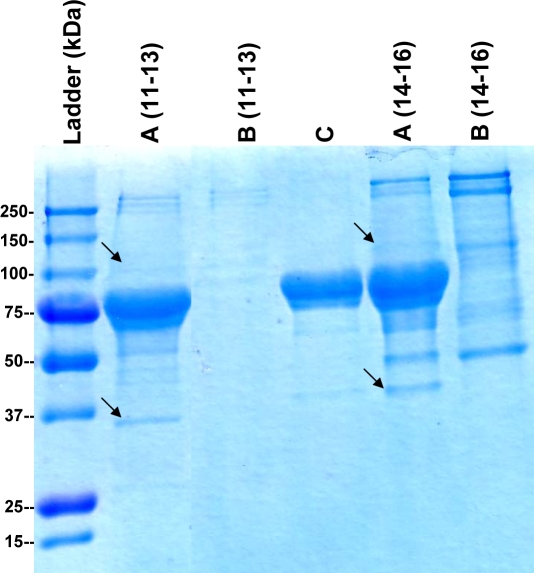
LDH-B and α-actinin 1 precipitate with GNE by *in vitro* binding assay. Gelcode reagent staining of in vitro binding assay eluates resolved by SDS-PAGE. Numbers (11–13, 14–16) correspond to anion-exchange pooled fractions. A, proteins bound to GNE-bound nickel beads; B, proteins bound to GNE non-bound nickel beads; C, control proteins bound to GNE-bound nickel beads. Black arrows point to either α-actinin 1 (∼100 kDa) or to LDH-B (∼37 kDa). Both proteins were identified by MS (Table 1).

In order to recognize the relevant proteins within the skeletal muscle lysate fractions which gave a positive GNE-binding signal, the pooled fractions (#11–13 and #14–16) were incubated with GNE-bound nickel beads. Two negative controls were used: Ni-NTA beads which were not bound to GNE were incubated with the same fractions, and GNE-bound Ni-NTA beads were incubated with lysis buffer. Bound proteins were eluted from the beads, resolved by a gradient SDS-PAGE and visualized by Gelcode reagent staining (Figure 2). The candidate bands, as well as the corresponding gel-areas or bands of the negative-controls, were cut from the gel for analysis by MS. Among all MS-identified proteins, only two were not found in negative controls. Those two proteins were identified as α-actinin 1 (103 kDa) and lactate dehydrogenase B (LDH-B) (36 kDa) (Table 1).

**Table 1:** MS identification of putative GNE binding partners.

Protein	MW kDa	Accession number	Sequence coverage (aa)	Matched peptides	Peptides sequences	Observed precursor m/z	z	Mr (expt.)	Mr (calc.)	Delta mass (amu)	Score
**LDHB**	36	gi|213255	12/334 (3.5%)	1/35	VIGSGTNLDSAR	595.2839	2	1188.5533	1188.6098	−0.0565	64
**alpha actinin 1**	103	gi|4501891	63/892 (7%)	5/103	LMLLLEVISGER	686.3339	2	1370.6533	1371.7795	−1.1262	97
					LVSIGAEEIVDGNVK	771.5139	2	1541.0133	1541.83	−0.8167	
					LASDLLEWIR	608.3039	2	1214.5933	1214.6659	−0.0726	
					LLETIDQLYLEYAK	856.4239	2	1710.8333	1710.908	−0.0747	
					VGWEQLLTTIAR	693.3639	2	1384.7133	1385.7667	−1.0534	

Bands from gradient SDS-PAGE (Figure 2) were digested by trypsin and analyzed by mass spectrometry through nano-ESI-MS-MS. For each protein, the following information is indicated: protein name, molecular mass (MW), accession number in NCBInr database, percentage of protein sequence covered by the matched and sequenced tryptic fragments, number of identified peptides out of the total number of tryptic peptides, peptides sequences, mass difference between experimental and theoretical masses, and score (aa, amino acids; expt., expected; calc., calculated).

### Biacore analysis with GNE and α-actinin 1

**Figure 3:**
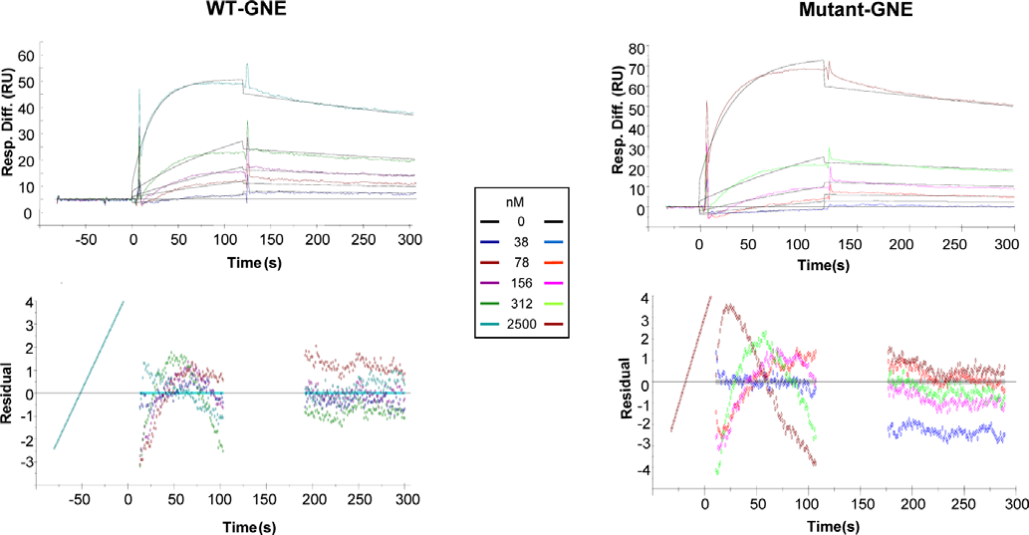
WT and mutant GNE proteins interact with α-actinin 1. Sensorgrams showing the interactions of GNE (WT and mutant) proteins with different α-actinin 1 concentrations and the residual curves, showing the fitness between the model and the experimental results [Resp. Diff., response difference; RU, response units].

The putative interaction between GNE and α-actinin 1 was tested directly using BIAcore. 15 µl of 5 µM α-actinin 1, diluted in Tris buffer containing 300 mM NaCl, were injected over immobilized GNE. Specific binding signals were obtained with both WT and mutant GNE proteins. The kinetics of these interactions were studied in order to determine their affinity and kinetics constants, and whether any differences in the affinity binding between WT and mutant GNE proteins could be detected. The results are shown on Figure 3 and Table 2. No consistent difference was obtained between the interactions of α-actinin 1 with WT compared to mutant GNE, in four independent assays. As a control for GNE-α-actinin 1 interaction specificity, 1–10 µM BSA were injected, and no interaction was detected. Since α-actinin 1 is calcium-sensitive [23], we analyzed the effect of CaCl_2_ on the interaction affinity between GNE and α-actinin 1, by replacing the 2 mM CaCl_2_ by 2 mM EDTA in the Tris buffer. Excluding CaCl_2_ decreased the affinity of both WT and mutant GNE to α-actinin 1 by ten fold (for WT, 6.8×10^−8^ M and 2.56×10^−7^ M, with and without CaCl_2_, respectively; for mutant, 4.5×10^−8^ M and 3.9×10^−7^ M, with and without CaCl_2_, respectively). The same result was obtained in two independent analyses, indicating that although this interaction is calcium independent in principle, calcium has a significant positive effect on it.

**Table 2:** Kinetics BIAcore analysis for the interaction of GNE with α-actinin 1.

	Ka (1/Ms)	Kd (1/s)	KD (M)	Chi^2^
**WT-GNE**	1.82×10^4^	1.24×10^−3^	6.8×10^−8^	0.619
**Mutant-GNE**	1.23×10^4^	1.01×10^−3^	8.2×10^−8^	1.58

Kinetics values of the interactions of WT and mutant GNE proteins with α-actinin 1 as calculated using 1∶1 Langmuir model.

### Biacore analysis with GNE and LDH-B

**Figure 4:**
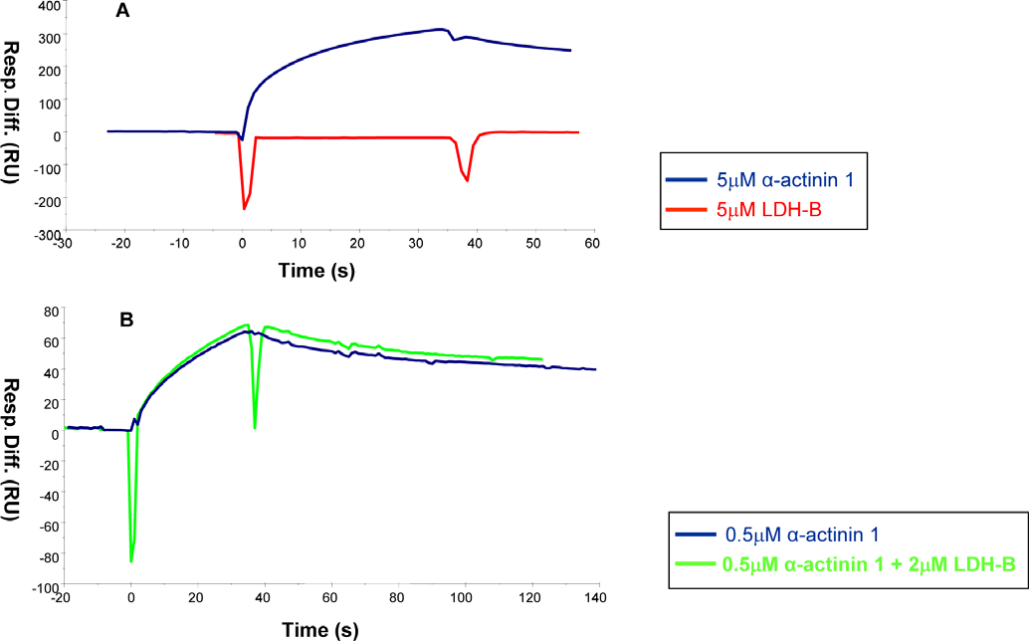
LDH-B does not show BIAcore binding signal with GNE. [A] Sensorgram of the interaction of GNE with 5 µM LDH-B, compared to 5 µM α-actinin 1. [B] Sensorgram showing the effect of 2 µM LDH-B on the interaction of GNE with 0.5 µM α-actinin 1 [Resp. Diff., response difference; RU, response units].

The putative interaction between GNE and LDH-B was tested as well. Various concentrations of LDH-B (1–27 µM) were injected in different buffers (Tris HEPES, PBS or NaPi), but no interaction was detected. Figure 4A shows the results obtained for α-actinin 1-GNE versus LDH-B-GNE interactions when tested at the same conditions: a binding signal was obtained with α-actinin 1, but not with LDH-B. As shown in Figure 4B, 2 µM LDH-B did not have any effect on the binding of 0.5 µM actinin to GNE, excluding the hypothesis that LDH-B may act as a co-factor for GNE-α-actinin 1 interaction. Therefore a direct interaction between GNE and LDH-B could not be demonstrated *in vitro* by BIAcore analysis.

### Co-Immunoprecipitation (co-IP) of FLAG-GNE and α-actinin

**Figure 5:**
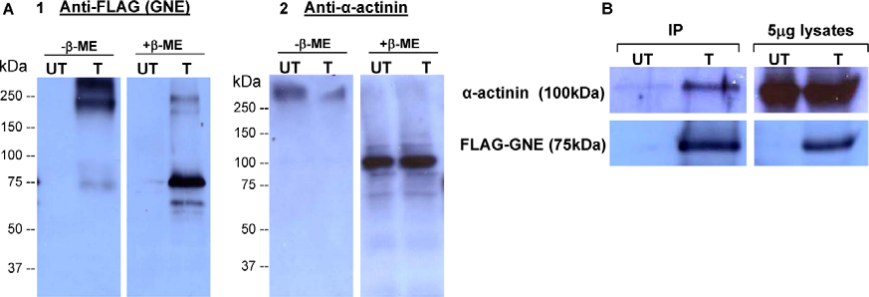
α-Actinin is co-immunoprecipitated with FLAG-GNE in 293T-FG cells. [A] Western blot analyses with anti-FLAG antibody (A1) and anti-α-actinin antibody (A2) on 5 µg lysate-samples of FLAG-GNE (FG)-transfected (T) and untransfected (UT) 293T cells, treated with SDS sample buffer in the presence (+) or absence (−) of β-ME. [B] Co-IP results. Western blot analyses with anti-FLAG antibody and anti-α-actinin antibody of IP eluates and 5 µg lysate-samples, of FLAG-GNE (FG)-transfected (T) and untransfected (UT) 293T cells.

To verify whether the interaction between GNE and α-actinin takes place also in a cellular environment, we performed co-immunoprecipitation experiments using human 293T cells, overexpressing the FLAG-GNE protein (293T-FG, or shortly T). As a negative control, we used untransfected 293T cells (UT) (Figure 5). α-Actinin is highly expressed in both cell types (Figure 5). By immunoprecipitation of cell protein lysates with anti-FLAG antibody bound beads, α-actinin was not detected with FLAG-GNE (data not shown). To test the possibility that the interaction is disrupted during the cell lysing process, proteins were crosslinked with DSP within the intact cells prior to their extraction. This crosslinker is membrane permeable, and can be cleaved by boiling the protein samples in 5% β-ME containing SDS-sample buffer. The effect of DSP crosslinking on both proteins was verified by western blot analyses with anti-FLAG and anti α-actinin antibodies (Figure 5A). Cell lysates (5 µg) were diluted in SDS-sample buffer, with or without 5% β-ME. In the absence of β-ME, the signal of both proteins was detected at the upper region of the SDS-PAGE gel, equivalent to MW of 250 kDa or higher, while β-ME treatment resolved the crosslinking and resulted in two distinct signals corresponding to the molecular weights of FLAG-GNE (75 kDa) (Figure 5A-1) and α-actinin (100 kDa) (Figure 5A-2). After immunoprecipitation with the anti-FLAG bound beads, the eluted protein samples were analyzed by western blot with anti-α-actinin and anti-FLAG antibodies. As shown in Figure 5B, both α-actinin and FLAG-GNE were detected only in the sample originating from 293T-FG cells, but not in UT control cells. Our results demonstrate that α actinin coimmunoprecipitates with FLAG-GNE in 293T-FG cells.

### Localization of GNE and α-Actinin 1 in muscle tissue

**Figure 6:**
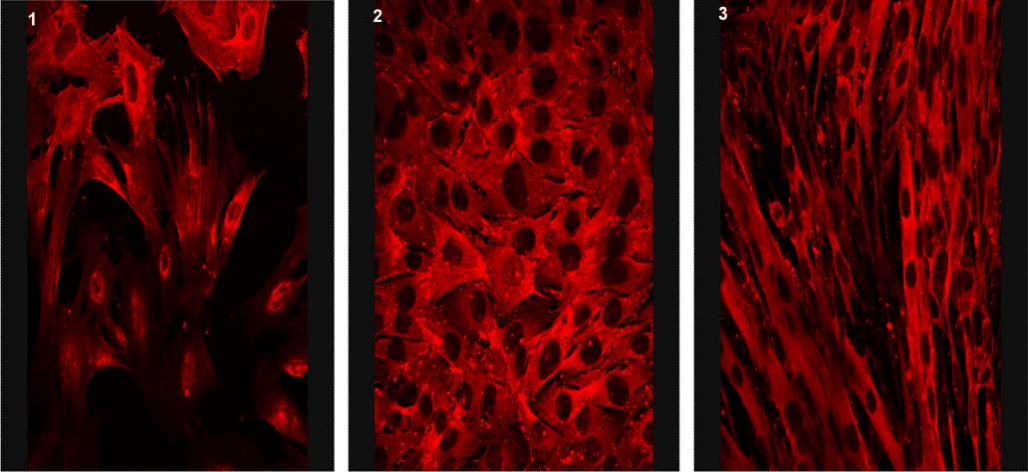
α-Actinin 1 is expressed in skeletal muscle. Light microscopy of mouse muscle culture (C2C12) at differentiation day (DD) 0 (1), DD 1 (2) and DD 3 (3) stained with antibody to α-actinin-1 (3A2).

**Figure 7:**
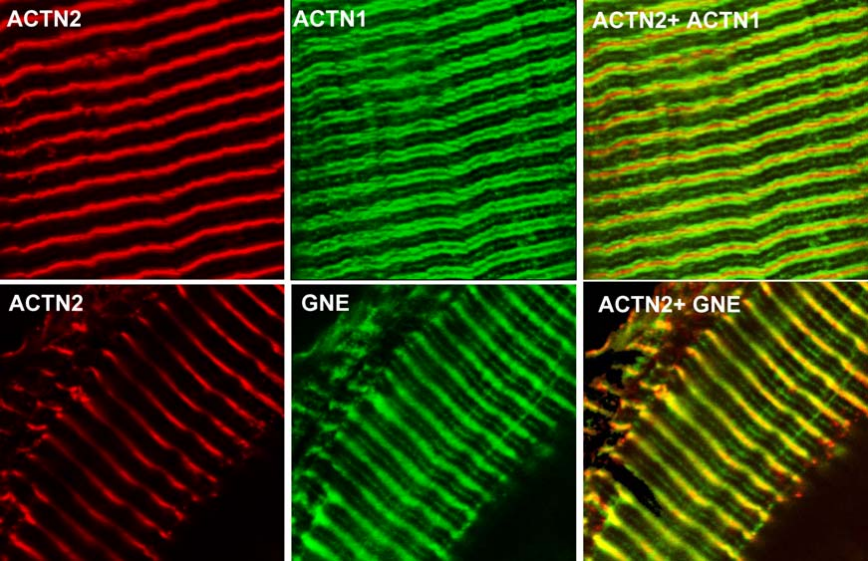
GNE and α-actinin 1 are expressed in stretched mouse muscle. Confocal microscopy of stretched mouse spinalis muscle stained with antibodies recognizing the Z line marker α-actinin-2 (ACTN2, red), α-actinin-1 (ACTN1, green), and GNE (GNE, green). α-Actinin-1 antibodies(3A2) and GNE antibodies show a distinct but overlapping staining pattern: GNE Abs recognize a diffuse band centered on the Z line while α-actinin-1 Abs stain two distinct bands on either side of the Z line. Both Abs recognize also, as a fainter staining, the sarcomeric M line. Overlap stain between α-actinin-1 and α-actinin-2 Abs is minimal.

α-Actinin 1 is generally regarded as one of the non-muscle isoforms of α-actinin family. To confirm that it is expressed in muscle cells rather than in non-muscle structures within muscles (such as blood vessels), we examined its expression in a mouse myoblast line (C2C12 cells). Indeed, α-actinin 1 antibody stained undifferentiated and differentiated myoblasts (Figure 6). To assess the expression of GNE and α-actinin 1 in mature muscle, we co-stained each of the polyclonal antibodies anti α-actinin 1 and anti GNE with an antibody to the muscle Z line marker sarcomeric actinin (α-actinin 2) in mouse stretched spinalis muscle (Figure 7). Both α-actinin1 (Figure 7A) and GNE (Figure 7B) antibodies recognized an overlapping but not fully superimposed striated pattern: antibodies to both proteins stained around or close to the muscle Z line, as well as a fainter staining at the sarcomeric M line. However the localization around the Z line differed between the 2 proteins in stretched muscle, with GNE forming a diffuse band centered on the Z line, while α-actinin 1 is found as two distinct bands on either side of the Z line, suggesting that GNE and α-actinin 1 are expressed in distinct but overlapping compartments in skeletal muscle.

## Discussion

The GNE gene is mutated in all HIBM patients [3], but the role of the altered GNE protein leading to the disease phenotype is not yet understood. The aim of our study was to search for potential GNE protein interactors which could unravel novel functions of GNE, possibly not related to its well known role in the biosynthesis of sialic acids. Since previous traditional co-immunoprecipitation assays for identification of GNE protein partners did not yield any positive results, we used BIAcore, an optical SPR-bio sensor (Surface Plasmon Resonance) system, to test GNE's interactions. BIAcore is generally used for kinetic characterization of a known interaction between two biomolecules. Since we had no clue about the identity of the potential GNE binding proteins in muscle cells, we applied the BIAcore system as a bioassay similar to the one described by Catimel et al. [24], who immobilized proteins onto the sensor chip and affinity purified binding partners from chromatographically enriched cell lysates. We immobilized WT and mutant (M712T) GNE proteins to the BIAcore sensor chip, and run through it anion exchanged fractions of human skeletal muscle primary culture cell lysate. Analysis of fractions showing binding signal by *in vitro* binding assay revealed two potential protein partners of GNE, LDH-B and α-actinin 1. While a direct interaction of GNE with LDH-B could not be shown by kinetics BIAcore analysis, this assay clearly illustrated the binding between GNE and α-actinin 1. Further, we showed by a co-IP assay that GNE and α-actinin interact in a cellular environment, and by immunohistochemistry, that GNE and α-actinin 1 are expressed in distinct but overlapping compartments in skeletal muscle.

α-Actinin 1 is one of the four family members of α-actinins (1–4), which belong to the spectrin protein superfamily including the spectrins and dystrophin [25]. α-Actinin is an actin binding and crosslinking protein, a homodimer with 100 kDa subunits which can be visualized as a long rod-shaped molecule by electron microscopy [23]. The α-actinins are known to be present in multiple subcellular compartments, including cell–cell and cell-matrix contact sites, cellular protrusions and stress fiber dense regions [26]. One of the protein isoforms, α-actinin 4, was found to be localized also to the nucleus in various cancer cell lines [27], [28], as well as in a differentiated skeletal muscle cell line [29]. GNE is an enzyme primarily localized in the cytosol (8). However, it was shown to be attached to the Golgi complex and in the nucleus of mammalian cells as well [22].

Binding of calcium to the non-muscle α-actinins (1 and 4) changes the conformation of the protein and inhibits its actin binding capacity [30]. In contrast, muscle α-actinins (2 and 3) are calcium insensitive since alternative splicing abolished their calcium binding ability [25]. Interestingly, we found that calcium has a significant positive effect on α-actinin 1 interaction with GNE. Further analysis is needed in order to recognize the precise site of interaction in both GNE and α-actinin 1, and to clarify how the conformational change caused by calcium binding improves this interaction.

The α-actinins contribute to both the stability and the plasticity of actin-based arrays [26]. In addition to their function as actin-crosslinking proteins, α-actinins have been found to interact with a large number of protein partners, and to play multiple roles in cells, including linking of the cytoskeleton to various transmembrane proteins, diverse signaling pathways, and regulating the activity of a variety of receptors [26]. In light of recent reports on the interaction in nerve cells between GNE and a brain specific protein involved in the modulation of the cytoskeleton, CRMP1 [31], and on the possible involvement of GNE in pAkt signaling pathways [16], the interaction of GNE with α-actinin 1 could point to the involvement of GNE in one or more of these processes.

Although the interaction of HIBM mutant GNE with α-actinin 1, as measured by BIAcore, could not be significantly distinguished from that of the wild-type protein, we cannot rule out the possibility that any micro-change in this interaction might contribute to HIBM pathophysiology. It might be that *in vivo* the mutant GNE binds to α-actinin 1 in a slightly different manner or affinity, and therefore the effect of this change is not seen in normal biological conditions, but it has a deleterious effect on HIBM cells upon long term stress conditions. Since the BIAcore *in vitro* assay cannot mimic *in vivo* stress conditions, further experiments in cellular environments, designed to test the differences between the interactions of mutant and WT GNEs with α-actinins, are needed in order to test this hypothesis. Another possibility of how the BIAcore *in vitro* conditions may conceal a difference between GNE wt and GNE M712T in actinin interaction may derive from the different oligomeric forms of the enzyme. GNE assembles in two oligomeric states, a tetramer with full enzymatic activity and a dimmer only possessing ManNAc kinase activity [32]. The relatively rough conditions for immobilization of recombinant GNE on the BIAcore chip most likely cause the decay of the tetramer and only dimmers are available in the interaction assay. A difference of tetrameric wt versus mutant GNE in actinin interaction can therefore not be excluded and should be analyzed by other methods, e.g. pull-down assays with pre-purified tetrameric or dimeric GNE. However, the possibility that tetrameric GNE cannot interact with actinin at all can be ruled out, as the conditions of the co-immunoprecipitation assay maintain the native, tetrameric state of the enzyme in cells.

In contrast to the sarcomeric isoforms of α-actinin (2 and 3), which are components of the sarcomeric Z disc where they crosslink actin filaments and stabilize the muscle contractile apparatus [25], α-actinin 1 and 4 are considered as the non-muscle isoforms of the protein. Therefore, we were surprised to find the GNE interactor expressed in skeletal muscle primary culture to be α-actinin 1. Interestingly, this is not the first evidence for the expression of non-muscle α-actinins in a myogenic cell line. Endo and Masaki [33] found that both skeletal and smooth muscle actinins are expressed in cultured embryonic chicken pectoralis cells. The two proteins were found at separate locations within the cells; skeletal muscle α-actinin was absent in myoblasts and was restricted to Z discs in myotubes. Smooth muscle α-actinin was diffused in the cytoplasm and on membrane-associated structures of myoblasts, and then confined to membrane-associated structures of myotubes. In contrast, Goffart et al. reported recently [29] that both α-actinin 1 and 4 are expressed in C2F3 cells, a subclone of the myogenic mouse C2C12 line. The two proteins were found to be expressed in both myoblasts and myotubes. α-Actinin 1 was present in the cytosol of both cell types, while α-actinin 4 was localized to both cytoplasm and nucleus of myoblasts, and mainly to the nucleus of myotubes. In the present study we have confirmed that α-actinin 1 is expressed in C2C12 muscle cultures, excluding the possibility that expression of α-actinin-1 in skeletal muscle is limited to smooth muscle structures such as blood vessels. To assess the relevance of our findings in mature muscle tissue, we examined the expression of α-actinin 1 and GNE in mouse stretched spinalis muscle. While the localization of the two proteins is distinct, a substantial degree of overlap provides preliminary evidence that the interaction observed *in vitro* may plausibly occur *in vivo* in mouse skeletal muscle. Further comprehensive investigation is needed to elucidate the precise localization of α-actinin 1 and GNE in the myofibrillar apparatus centered on the Z line, and the relevance of α-actinin 1 function in skeletal muscle to its interaction with GNE in general, and to HIBM pathophysiology in particular.
